# Port Site Placement and Outcomes for Surgical Obesity and Metabolic Surgeries (PSPOSO) Checklist: A New Reporting Checklist Based on Evidential Assessment of the Number of Trocars and Positions

**DOI:** 10.1007/s11695-025-07694-y

**Published:** 2025-02-04

**Authors:** Mohamed H. Zidan, Hassan El-Masry, Ahmed Amgad, Hashem Altabbaa, Marwan Emad Abdou, Samar A. Amer, Nour Zayed, Haidy Osama Ismail, Mohammed Alokl, Ahmed Abokhozima

**Affiliations:** 1 Alexandria University, Alexandria, Egypt; 2 The Research Papyrus Lab, Alexandria, Egypt; 3 Helwan University, Cairo, Egypt; 4 Zagazig University, Zagazig, Egypt; 5 El-Ekbal Hospital, Alexandria, Egypt; 6 Department of Surgery, Medical Research Institute, Alexandria, Egypt

**Keywords:** Laparoscopic sleeve gastrectomy, Trocar placement, Metabolic and bariatric surgeries, Port site ergonomics, PSPOSO checklist, Surgical outcomes, Manipulation angles, Excess weight loss

## Abstract

Since the early 1980s, efforts to standardize ergonomic practices in laparoscopic surgeries have aimed to improve procedural efficiency and reduce complications, but clinical validation remains limited. In metabolic and bariatric surgeries (MBS), innovations in trocar site placements, driven by the popularity of laparoscopic sleeve gastrectomy (LSG), have advanced surgical techniques. However, practices often vary based on individual surgeon preferences rather than standardized evidence-based criteria. This study introduces the Port Site Placement and Outcomes for Surgical Obesity and Metabolic Surgeries (PSPOSO) checklist, aiming to standardize port placements and improve reporting consistency. A systematic review and meta-analysis of LSG studies were conducted following PRISMA guidelines. Data were extracted from 34 studies involving 7173 cases. Key variables included port configurations, manipulation angles, and outcomes such as operative time and excess weight loss percentage (EWL%). Innovative methods were used to estimate manipulation and azimuth angles from available intraoperative images. Statistical analyses and meta-regression were performed to identify associations between port configurations and surgical outcomes. Findings revealed substantial variability in port placements, with no significant effect of manipulation angles or port numbers on operative time or EWL% at 6, 12, and 24 months (*p*-values > 0.05). High residual heterogeneity suggests that factors beyond manipulation angles and port counts contribute to outcome variability. The PSPOSO checklist provides a framework for standardizing port placement and ergonomic parameters in MBS, enhancing reproducibility and safety. Future studies should validate the checklist across diverse clinical settings to refine surgical approaches and improve patient outcomes. PROSPERO: CRD42024598674.

**Supplementary Information:**

The online version contains supplementary material available at 10.1007/s11695-025-07694-y.

## Introduction

Since the inception of laparoscopic surgeries in the early 1980s, considerable efforts have been made to establish a standardized ergonomic approach to abdominal procedures [1]. Various ergonomic parameters, including manipulation angles and azimuth angles, have been developed to support the slow learning curve for surgeons, facilitating procedural efficiency and aiming to reduce complications [2, 3]. Although these parameters are widely recognized within the scientific community, they have generally lacked validation in clinical settings, contributing to the absence of a consistent validation framework in surgical literature. This inconsistency has led to procedure-specific adaptations, creating disparities, differing idealizations, and unique learning curves for each technique.

As minimally invasive surgery became integral across procedures, the need emerged for well-positioned port sites tailored to procedural steps, technical demands, and ergonomic requirements for the surgeon. In response, diverse methodological structures have evolved to optimize surgical techniques, decrease operative time, and reduce both patient comorbidities and surgeon discomfort [4].

In the realm of metabolic and bariatric surgeries (MBS), surgeons have honed their skills through similar learning curves, with different facilities prioritizing specific outcomes. For example, some have concentrated on improving the cosmetic appearance of trocar sites, while others have emphasized safety and reduced operative time without compromising procedural steps. Ultimately, each surgeon’s focus has been on delivering optimal patient outcomes through their techniques. Furthermore, some studies have lacked scientific evidence to provide these idealizations; rather, separate reports to establish ideal trocar placements were conducted that did not depend on scientific evidence [5].

Laparoscopic sleeve gastrectomy (LSG) has significantly influenced the development of versatile trocar sites due to its procedural feasibility and the increasing number of patients undergoing this surgery [6]. Thus, LSG has spurred the development of innovative trocar site placement positions, such as trans-umbilical single-port sleeve gastrectomy (SG) [7], bikini-line SG [8], and three-port SG [9]. Consequently, many surgeons have significantly advanced their learning curve not only for LSG but also for other more complicated MBS [10] and upper gastrointestinal surgeries [11].

Since the advent of laparoscopic surgeries, various ergonomic parameters, such as manipulation and azimuth angles, have been developed to help surgeons navigate the learning curve in minimally invasive procedures [12]. While these parameters are acknowledged within the scientific community [7], they have often lacked clinical validation, resulting in an absence of a standardized system in the surgical literature. Ergonomics in laparoscopy is designed to make procedures more efficient, reduce operative time, minimize complications, and improve outcomes. Yet, ergonomic parameters remain underrepresented in literature, prompting us to use a computerized systematic analysis to produce a preliminary evidential basis for these variables. This approach aims to introduce a new reporting checklist that supports future research and fosters greater consistency regarding the ergonomic benefits of specific trocar positions for patient outcomes.

Advances in these surgical approaches have motivated surgeons to share their methods widely, allowing further evaluation through secondary research. However, a universal framework for trocar site placement is still lacking, and many studies do not consistently address the ergonomic aspects, surgeon positioning, or critical outcomes like operative time, excess weight loss percentage (EWL%), and comorbidity developments, such as in Shah et al.’s report [5]. This absence of a standardized basis often results in practices based on individual preference or institutional custom rather than on objective, evidence-based criteria.

Our proposed PSPOSO (Port Site Placement and Outcomes for Surgical Obesity and Metabolic Surgeries) checklist responds to this need by setting standardized criteria for port location, manipulation angles, and patient positioning. Through this framework, we aim to reduce variability in MBS, enable clearer outcome comparisons, and provide consistency in reporting key surgical parameters. PSPOSO thus addresses an existing gap by offering a structured approach to support uniformity and reproducibility in port placement practices across diverse clinical settings.

This systematic review and meta-analysis aims to analyze the current literature on LSG ergonomics, establishing a consensus on optimal port site selections and ergonomic considerations for bariatric procedures. Although surgeons should select trocar sites based on their expertise, learning curve, and patient-specific needs, there remains a strong need for a unified language to enhance evidence-based approaches. Here, we provide a preliminary PSPOSO checklist as a foundational reporting tool for trocar site placement and ergonomics in MBS, serving as an evidential baseline to guide future research and improve standardization across the field.

## Methods

### Search Strategy

We conducted this systematic review and meta-analysis with a literature search of relevant studies on LSG, following the PRISMA guidelines [13] using PubMed, Scopus, Embase, and Cochrane databases. Additionally, we manually added one paper through supplementary search efforts. The MESH terms used in our search were (bariatric surgery OR Sleeve gastrectomy OR vertical sleeve gastrectomy) And (Laparoscope OR Laparoscopic) AND (Ports OR Port site OR Trocar placement OR Number of Trocars). The study was registered in PROSPERO and obtained a reference number of CRD42024598674.

**Fig. 1:**
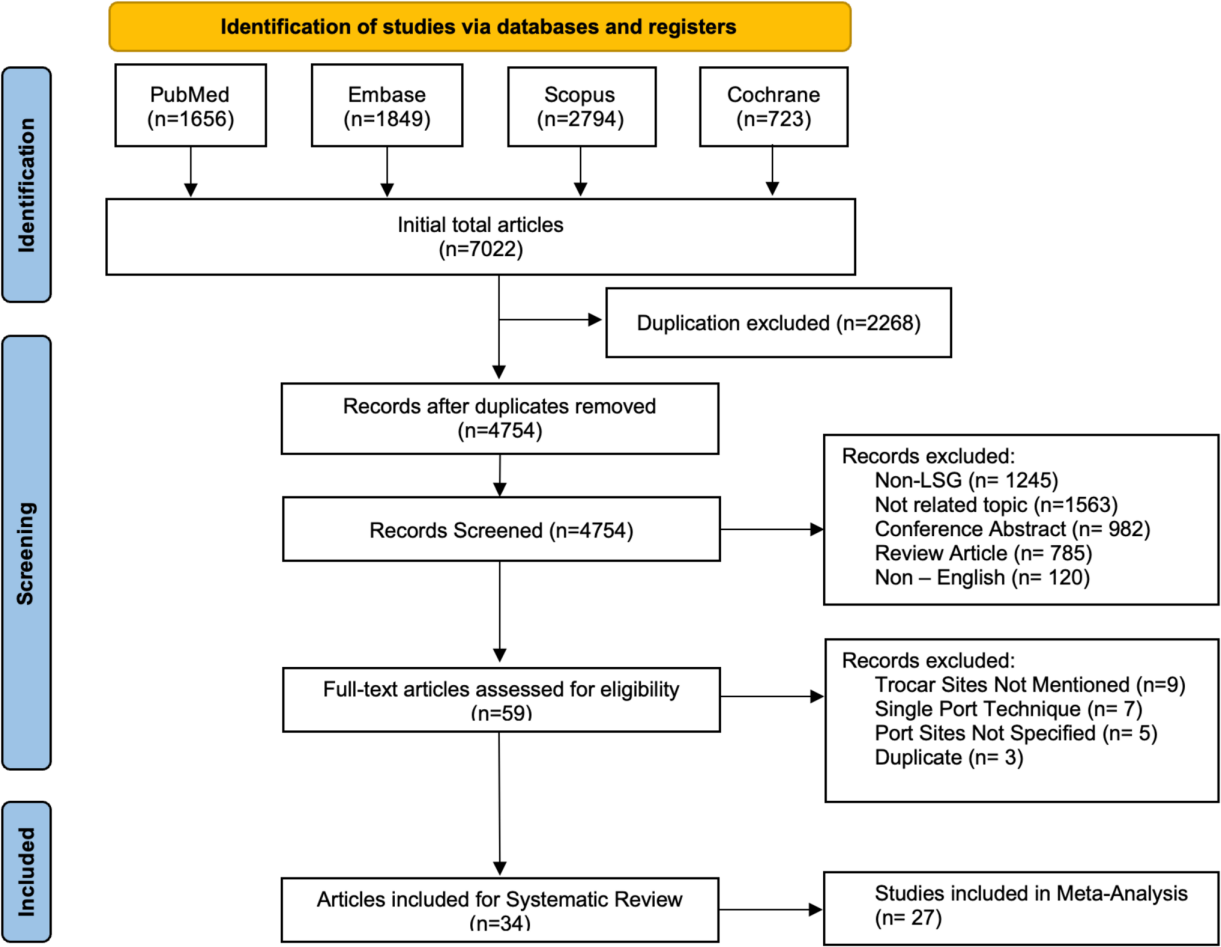
A PRISMA flow chart showing the studies included in both systematic review and meta-analysis

A total of 7022 publications were identified from the search, and one paper was manually added through supplementary efforts. After the initial search, EndNote was used to remove duplicates, followed by title and abstract screening and full-text screening. Exclusion criteria involved other bariatric procedures apart from LSG, non-English texts, review articles, case reports and case series, non-related topics, conference abstracts, and data that did not explicitly address trocar sites. Data extraction was carried out by two different authors, followed by validation by the same authors in different studies. Two different authors then performed revalidation for the extracted data. We included 34 relevant studies [14–46] with 7173 cases for analysis. The PRISMA flow diagram (Fig. 1) details our search methodology.

### Identified Variables

In our analysis, we gathered a comprehensive array of variables pertaining to trocar placement from the studies we examined. These variables encompassed the number of ports utilized, the location and dimensions of each optical port (trocar 1), as well as the right and left working hand sites (Ports 2 and 3) and their respective sizes. We also documented the locations and sizes of Ports 4 through 7. Additionally, we recorded the patient-positioning techniques on the operating table (straight-leg or open-leg position), and whether the port sites were sectorized or triangulated. Furthermore, we included variables such as age, gender, preoperative BMI, operative time, and percentage of excess weight loss (EWL%) at 6, 12, and 24 months in our analysis.

### Calculations of the Working Angles

According to the literature, the working angles are the angles used during surgical manipulation through port sites; these angles include the manipulation angle, the azimuth angle, and the elevation angle. As there were no recordings of the working angles in any of the studies, we developed an innovative method to estimate these angles. Some of the papers we analyzed contained intraoperative photographs or illustrations of port site locations, while others detailed trocar placement of the abdomen in the operative details sections without visual aid.

For studies with available visual data, we used ImageJ software to measure the manipulation angles (angles between both working hands) and the right and left azimuth angles (angles between the working hands and the optical trocar).

For consistency in reporting, we designated the right azimuth angle as “azimuth angle R x,” where “R” indicates the right-side angle and “x” corresponds to the patient number (e.g., azimuth angle R1, azimuth angle R2). Similarly, the left azimuth angle is identified as “azimuth angle L x” following the same nomenclature. We referred to the mean of the azimuth angles as “mean R azimuth angles” and “mean L azimuth angles” for the depiction of statistical analysis.

In cases where visual data was not available, we generated figures based on operative details from the articles and positioned the port sites on a template figure (Supplementary File 1). These figures were refined using Adobe Photoshop and then analyzed the angles using ImageJ software.

To calculate the angles, we utilized Adobe Photoshop to create virtual lines from the trocar’s insertion points to a consistent center point based on the surface anatomy of the hiatus. Three lines were drawn: one from the optical port to the hiatus, one from the right working hand to the hiatus, and one from the left working hand to the hiatus. These lines were then used to determine azimuth angle 1 (the angle between the optical port and the right-hand working port), azimuth angle 2 (the angle between the optical port and the left-hand working port), and the manipulation angle (the angle formed by the right-hand and left-hand working ports) using ImageJ software for Windows.

Given the challenges in estimating the elevation angle—due to factors such as the height of the operating table, the length of the surgical instruments, and the patient’s body habitus—we opted to focus exclusively on the manipulation and azimuth angles for our analysis.

The reference meeting point for LSG was determined to be the hiatus, as it represents the furthest point of the surgical maneuver. It is important to note that the calculated angles were estimates for this furthest point, and the dissection of the lowest part of the greater curvature would likely involve different angles. Our technique did not address this limitation, as the variation in stomach size among patients was not accounted for. Additionally, the estimated calculations were provided as a mean estimate for each study and not tailored to individual patients, resulting in increased heterogeneity in the data. Nevertheless, this methodology represents an innovative approach to establishing standardized reporting fundamentals for trocar sites to mitigate such heterogeneity. We credit the conceptualization and formulation of this methodology to MHZ and NZ respectively.

### Risk of Bias

The methodological quality of the studies included in the review was evaluated using the Newcastle–Ottawa Scale (NOS), which examines three key domains: participant selection, comparability of study cohorts, and outcome assessment. Each study underwent independent assessment by two reviewers, with any disagreements resolved either through consensus or by involving a third reviewer. The NOS framework classifies studies of low, intermediate, or high risk of bias, thereby providing a consistent and objective measure of quality.

### Data Analysis

We used R software version 4.4.1 to perform a comprehensive analysis using the (Meta) and (Metafor) packages. Our focus was on assessing operative time and EWL% across single-arm intervention studies. Since effect sizes could not be calculated, we instead conducted a subgroup analysis of 29 studies, categorizing them according to the number of ports used.

For our analysis, outcomes such as operative time and EWL% at 6, 12, and 24 months were evaluated. Where standard deviations (SDs) were not reported, we estimated them by dividing the range by four and derived variance from these approximated SDs. Following the subgroup analysis, we performed meta-regression to explore the impact of variables such as BMI, manipulation angles, right-handed angles, and left-handed angles on operative time.

The meta-regression analysis was conducted using the (Metafor) package, with the following assumptions and values considered: estimates of the regression coefficients (*β*), standard errors (SE), *Z*-values, *p*-values, and confidence intervals. The statistical model included these variables as predictors to assess their effect on the outcomes. A *p*-value of 0.05 was considered statistically significant. Heterogeneity within subgroups was assessed using the *I*^2^ statistic to gauge the variability between studies, with *I*^2^ values indicating moderate to high heterogeneity.

## Results

### Systematic Analysis

The systematic review includes data from 35 studies, covering a total of 7173 cases. Baseline characteristics such as age, sex, preoperative body mass index (BMI), operative duration, and average preoperative BMI across surgeries with three, four, five, six, and seven ports.

Outcomes, including operative time and EWL% at 6, 12, and 24 months, are summarized in Table 1. The average age of participants is 39.65 ± 5.04 years. Sex distribution indicates that 29.1% of the participants were male, 67.8% were female, and 3.1% had unreported gender in the studies [19, 30]. The preoperative BMI averages 44.71 ± 5.97 kg/m^2^, with BMI reported separately for each group according to port numbers (Table 2).

**Table 1:** Summary of all the studies included in the systematic review

Author	Country	No. of ports	No. of cases	Mean Age (years)	Sex (F/M)	Mean Preoperative BMI (kg/m^2^)	Mean Operative time (min)	Mean EWL% at 6 months	Mean EWL% at 12 months	Mean EWL% at 24 months
Roa PE, et al	USA	7	30	40	23/7	41.4	80	52.8	_	_
Moy J, et al	USA	7	135	43.5	75/60	60.1	134	39.9	47.3	_
Fuks D, et al	France	4	135	40	113/22	48.8	103	38.6	49.4	56
Chowbey PK, et al	India	5	75	44.4	40/35	58.0	60	52.3	59.13	65.2
Bellanger DE, et al	USA	5	529	43.43	431/98	44.26	_	42.36	65.92	66.11
D’Hondt M, et al	Belgium	6	83	40.4	61/22	39.3	_	_	78.5	72
Angrisani L, et al	Italy and USA	5	127	38.8	78/49	48.7	105	48.1	51.7	53.1
Chopra A, et al	USA	4	174	39.59	149/25	48.97	103.9	44.76	55.52	59.22
Gadiot RP, et al	Netherlands	5	445	42	335/110	46	41	_	71	69
Prasad P, et al	India	5	110	39.3	84/26	44.58	64.81	53.15	67.57	71.19
Noel P, et al	France	3	750	41.2	570/180	43.7	_	59	73	73
Zachariah SK, et al	Taiwan	5	228	34.68	145/83	37.42	60.63	_	72.39	72.63
Gibson SC, et al	Australia	4	500	41	340/160	45	_	58	76	71
Schraibman V, et al	Brazil	5	32	46	16/16	39.4	138	_	_	_
Park JY, et al	Korea	6	192	33.1	131/61	40.0	104.4	60.8	72.6	80.6
Seki Y, et al	Japan	5	179	40.7	89/90	43.3	140	_	68.5	72.9
Obeidat F, et al	Jordan	5	190	34	_	46.2	90.4	60.1	75.1	72.6
Yildiz B, et al	Turkey	5	159	39.6	131/28	47.5	105	53.1	70.1	75.1
Al-Mulhim AS	KSA	4	112	26	73/39	41	151	_	43	_
Sepúlveda M, et al	Chile	4	1023	40.6	688/335	37	67.6	_	_	_
Bhandari M, et al	India	6	152	40.4	81/71	45.0	46	_	_	_
Lemaître F, et al	France	5	494	46.2	367/127	60	60	_	31.5	31.3
Biter LU, et al	Netherlands	5	76	45.5	63/13	44.17	_	_	73.21	_
Lakdawala M, et al	India	5	300	35.5	150/150	39.9	42	58.6	68.3	69.1
Porta A, et al	Italy	5	65	39	51/14	41.01	61	48.3	56.5	59.9
Morales-Conde S, et al	Spain	5	15	46.8	15/0	45.52	61.80	53.3	_	_
Mauriello C, et al	Italy	3	187	52	106/81	53.3	72	59.7	65.1	67.2
Amiki M, et al	Japan	5	31	39.7	_	33.7	120.2	_	109.7	105.3
Tranchart H, et al	France	4	314	41	246/68	42.7	50	73.3	73.4	_
Sucher R, et al	Austria	4	40	43	0/40	43.8	97.4	_	_	_
Muir KB, et al	USA	5	30	35	27/3	46	90.7	48.3	_	_
Bang GA, et al	Cameroon	5	21	40.3	19/2	44.9	192.2	_	38.89	_
Abdelsamee KS, et al	Egypt	5	70	32.07	54/16	42.62	53.01	_	_	_
Abdelsamee KS, et al	Egypt	3	70	29.26	59/11	41.68	51.81	_	_	_
Pan HM, et al	Taiwan	3	100	38.8	52/48	39.4	72.5	_	_	_

**Table 2:** Demographic data included in the systematic review

Demographic data	*N* (%) Mean ± SD
**Number of**	
Studies	35
Cases in studies	7173
**Age**	
Min–max	17–76
Mean ± SD	39.79 ± 5.12
**Sex**	
Male	2090 (29.1)
Female	4862 (67.8)
**Preoperative BMI (kg/m^2)**	
Min–max	30–90
Mean ± SD	44.71 ± 5.97
**Preoperative BMI (kg/m^2)**	
3 trocars subgroup	44.52 ± 4.29
4 trocars subgroup	43.89 ± 6.38
5 trocars subgroup	44.93 ± 5.77
6 trocars subgroup	41.43 ± 7.8
7 trocars subgroup	50.75 ± 10.6

#### Operative Details

The intraoperative mean operative time was 87.31 ± 37.10 min. Trocar positions were determined by aligning the optical port with the working ports, with 88.6% arranged in triangulation and 11.4% in sectorization. Port numbers varied: 44.3% of studies used 5 trocars, 32% used 4, 15.4% used 3, and 8.3% used more than 5, selected based on the trocar’s role during dissection.

The optical port was labeled “Port 1,” the right working hand trocar as “Port 2,” the left working hand trocar as “Port 3,” the liver retraction trocar as “Port 4,” and the assistant trocar as “Port 5.” Some studies mentioned additional trocars without specifying their functions. For studies using 4 trocars, configurations included either 4 with an assistant port or 4 with a liver retraction port. In studies with only 4 trocars and an assistant port, “Port 5” represented the assistant port, with “Port 4” omitted. Placement for “Port 1” was predominantly peri-umbilical (77.1%), “Port 2” in the left subcostal quadrant (62.6%), and “Port 3” in the right subcostal quadrant. Mean port sizes for the optical, right working hand, and left working hand ports were 10.66 mm, 9.97 mm, and 11.29 mm, respectively, while “Port 4” and “Port 5” showed varied placements, including sub-xiphoid and right subcostal locations (Table 3).

**Table 3:** Intraoperative data records extracted and calculated from the studies included in the systematic review

Intraoperative data	*N* (%) Mean ± SD *n* = 7173
**Operative time (min)**	
Min–max	19–550
Mean ± SD	87.31 ± 37.10
**Number of ports (trocars)**	
Min–max	3–7
Mean ± SD	4.81 ± 0.98
**Number of ports (trocars)**	
3 trocars	1107 (15.4)
4 trocars	2298 (32)
5 trocars	3176 (44.3)
6 trocars	427 (6)
7 trocars	165 (2.3)
**Optical port (Port 1) site**	
Peri-umbilical	5530 (77.1)
Trans-umbilical	409 (5.7)
Left subcostal min-clavicular line	409 (5.7)
Left mid-clavicular line	409 (5.7)
Right hypochondrium	208 (2.9)
Left lower abdomen	208 (2.9)
**Size of optical port (Port 1)**	
Min–max	5–15
Mean ± SD	10.66 ± 2.26
**Right working hand (Port 2) site**	
Left subcostal	4512 (62.9)
Left mid-clavicular at the level of the umbilicus	409 (5.7)
Midline at trans tubercular plane	409 (5.7)
Left anterior axillary	609 (8.5)
Left mid-clavicular	818 (11.4)
Left hypochondrium	208 (2.9)
Umbilical	208 (2.9)
**Size of Port 2**	
Min–max	5–15
Mean ± SD	9.97 ± 3.79
**Left working hand (Port 3) site**	
Right subcostal	4512 (62.9)
Epigastric port	409 (5.7)
Right mid-clavicular at the level of the umbilicus	1628 (22.7)
Left subcostal	208 (2.9)
Supra-umbilical	208 (2.9)
Right upper quadrant	208 (2.9)
**Size of Port 3**	
Min–max	5–15
Mean ± SD	11.29 ± 3.15
**Site of Port 4 (assistant port)**	
Left axillary	3070 (42.8)
Left subcostal	1227 (17.1)
Right mid-clavicular at the trans-iliac line	208 (2.9)
Right costal margin	208 (2.9)
Right pararectal	208 (2.9)
Subxiphoid	208 (2.9)
Not mentioned	2044 (28.5)
**Size of Port 4**	
Min–max	2–12
Mean ± SD	5.44 ± 1.80
**Site of Port 5 (liver retraction port)**	
Sub-xiphoid/upper epigastric port	4921 (68.6)
Right subcostal port	1227 (17.1)
Not mentioned	1025 (14.3)
**Size of Port 5**	
Min–max	2–12
Mean ± SD	6.28 ± 2.91
**Site of Port 6**	
Right mid-clavicular mid lumbar	208 (2.9)
Right mid-epigastric	208 (2.9)
Right anterior axillary	208 (2.9)
Right subcostal	208 (2.9)
Left mid-clavicular at the trans-iliac line	208 (2.9)
Not mentioned	6133 (85.5)
**Size of Port 6**	
Min–max	5–15
Mean ± SD	9.50 ± 5.05
**Site of Port 7**	
Epigastric port below sub-xiphoid	208 (2.9)
Right high epigastrium	208 (2.9)
Not mentioned	6757 (94.2)
**Size of Port 7**	
Min–max	5–5
Mean ± SD	5.00 ± 0.0
**Patient position on the operating table**	
European position	5537 (77.2)
American position	818 (11.4)
Not mentioned	818 (11.4)
**Triangulation vs sectorization of trocars**	
Triangulation	6355 (88.6)
Sectorization	818 (11.4)

Patient positioning on the operating table was categorized as open-leg or straight-leg positions. The open-leg position involved a position in anti-Trendelenburg, with the surgeon standing between the patient’s legs, while the straight-leg position had the patient inclined slightly to the right side in anti-Trendelenburg, with the surgeon standing on the right. Among the studies, 77.2% used the open-leg position, 11.4% the straight-leg position, and 11.4% did not specify.

#### The Estimated Working Angles

We calculated the extracted angles from ImageJ (Table 4). The estimated manipulation angle had a mean of 83.05 ± 25.89 degrees. The R azimuth angle (between “Port 1” and “Port 2”) had a mean of 45.32 ± 14.57, while the L azimuth angle (between “Port 1” and “Port 3”) had a mean of 44.39 ± 18.57.

**Table 4:** Estimated calculated angles from the studies included in the systematic review data

Angles	*N* (%)
**Estimated manipulation angle**	
Min–max	28.48–125.04
Mean ± SD	83.05 ± 25.89
**Estimated R azimuth angle**^**a**^	
Min–max	22.76–69.66
Mean ± SD	45.32 ± 14.57
**Estimated L azimuth angle**^**b**^	
Min–Max	16.23–95.40
Mean ± SD	44.39 ± 18.57

^a^R azimuth angle = azimuth angle between “Port 1” and “Port 2”

^b^L azimuth angle = azimuth angle between “Port 1” and “Port 3”

### Risk of Bias

**Fig. 2:**
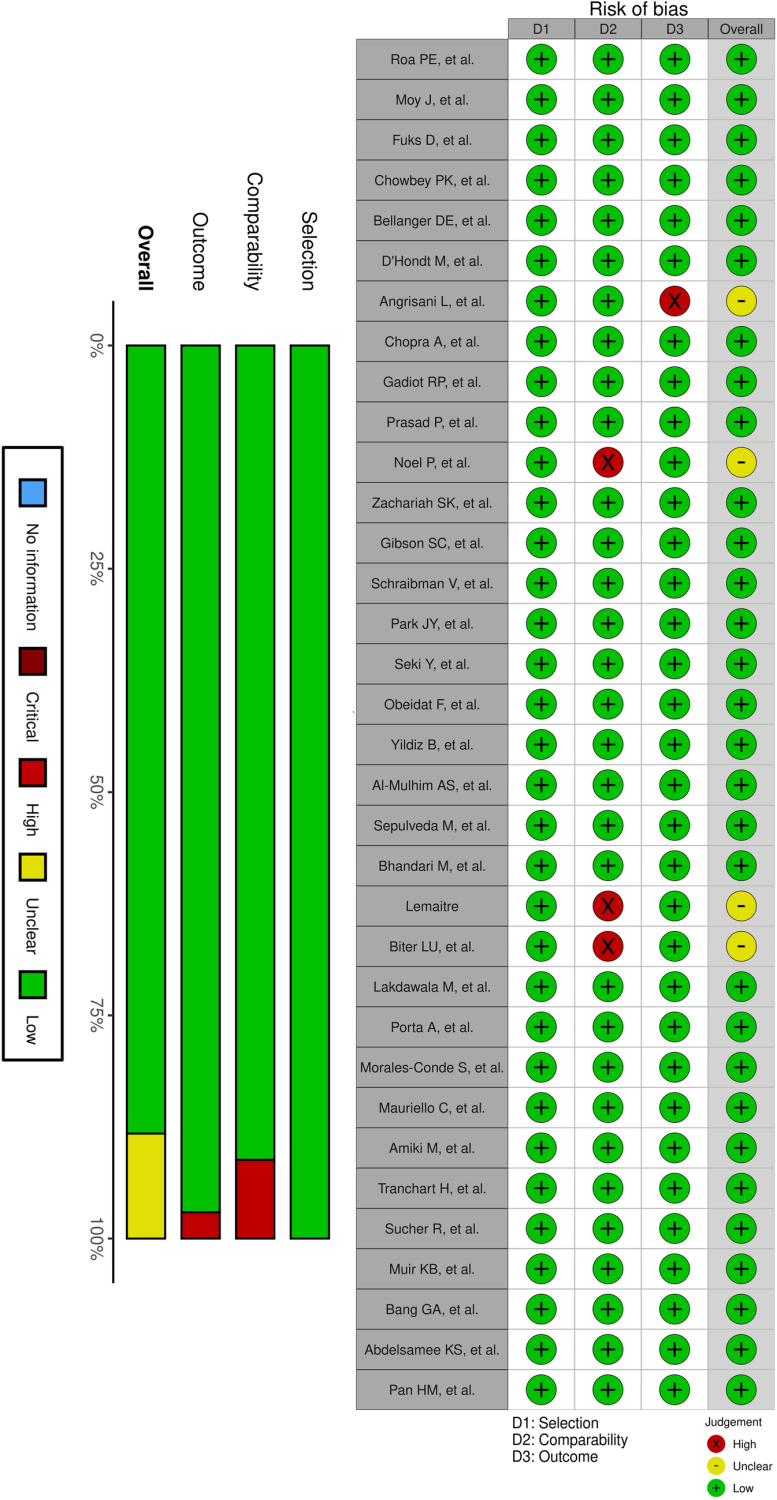
Review authors’ judgments about each risk of bias domain for each included study using the Newcastle–Ottawa Scale (NOS)

The quality assessment indicated that most studies achieved a low risk of bias in the selection and outcome domains, demonstrating well-structured methodologies and rigorous outcome reporting (Fig. 2). These studies followed appropriate protocols in participant selection, ensuring minimal selection bias, and applied consistent outcome measures. However, the comparability domain exhibited some variability, with a subset of studies rated as having intermediate or high risk of bias, primarily due to inconsistencies in cohort matching or insufficient control of confounding variables. These discrepancies suggest that while the internal validity of most studies was strong, a few investigations may have been affected by differences in baseline characteristics between groups, which could influence their findings. Overall, the included studies were of high methodological quality, with the majority meeting the criteria for low bias across key domains.

### Meta-analysis

#### Operative Time

**Fig. 3:**
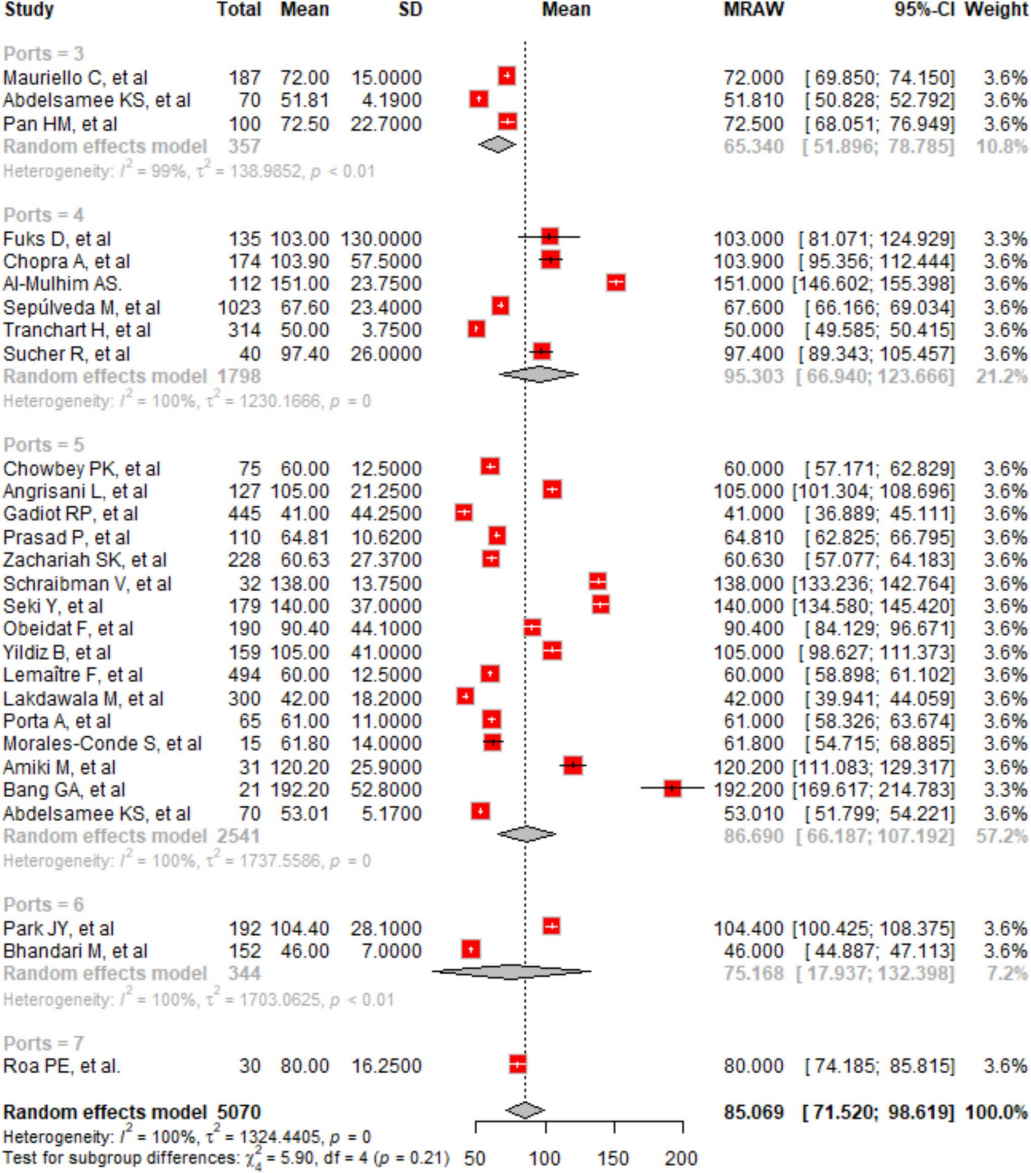
The forest plot shows the overall mean operative time and variations across studies and subgroups, highlighting confidence intervals, and variability

A meta-analysis of 28 studies with 5070 cases evaluated the mean operative time across laparoscopic procedures. The overall mean operative time was 85.07 min (95% CI, 71.52 to 98.62), based on a random effects model (Fig. 3). In subgroup analyses by port number, the mean operative times were 80.00 min for seven ports (95% CI, 74.19 to 85.81), 95.30 min for four ports (95% CI, 66.94 to 123.67), 86.69 min for five ports (95% CI, 66.19 to 107.19), 75.17 min for six ports (95% CI, 17.94 to 132.40), and 65.34 min for three ports (95% CI, 51.90 to 78.78). Each subgroup displayed high heterogeneity (*I*^2^, 99.4 to 99.9%), with no significant difference in operative times across different port numbers (*Q* = 5.90, d.f. = 4, *p* = 0.2069). The Egger’s test indicated significant asymmetry (*p* < 0.0001), suggesting potential publication bias.

### Excess Weight Loss Percentage

The relation between the number of ports and EWL% was analyzed at 6, 12, and 24 months.

#### EWL% at 6 Months

**Fig. 4:**
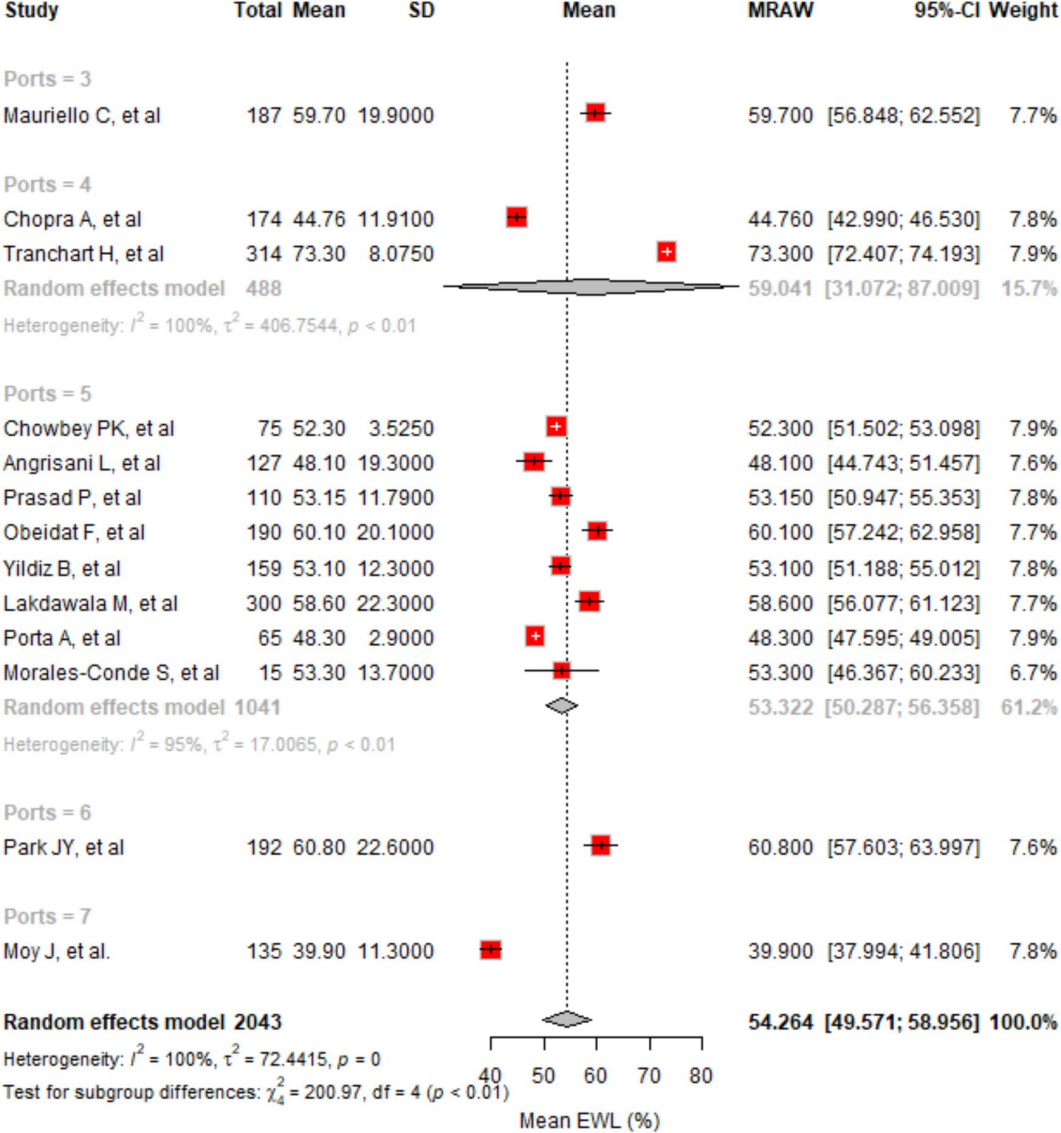
The forest plot displays the mean EWL% at 6 months across different studies, segmented by the number of ports used

Analyzing 13 studies with 2043 cases, the average EWL% at 6 months was 54.26% (95% CI, 49.57–58.96%) using a random effects model (Fig. 4). High variability was present (*I*^2^ = 99.5%), with subgroup analysis indicating different EWL% by port number: 59.70% for 3 ports, 59.04% for 4 ports, 53.32% for 5 ports, 60.80% for 6 ports, and 39.90% for 7 ports. The differences across port groups were statistically significant (*p* < 0.0001). The 6-port approach had the highest EWL%, and the 7-port had the lowest. Egger’s test showed no significant publication bias (*p* < 0.0001).

#### EWL% at 12 Months

**Fig. 5:**
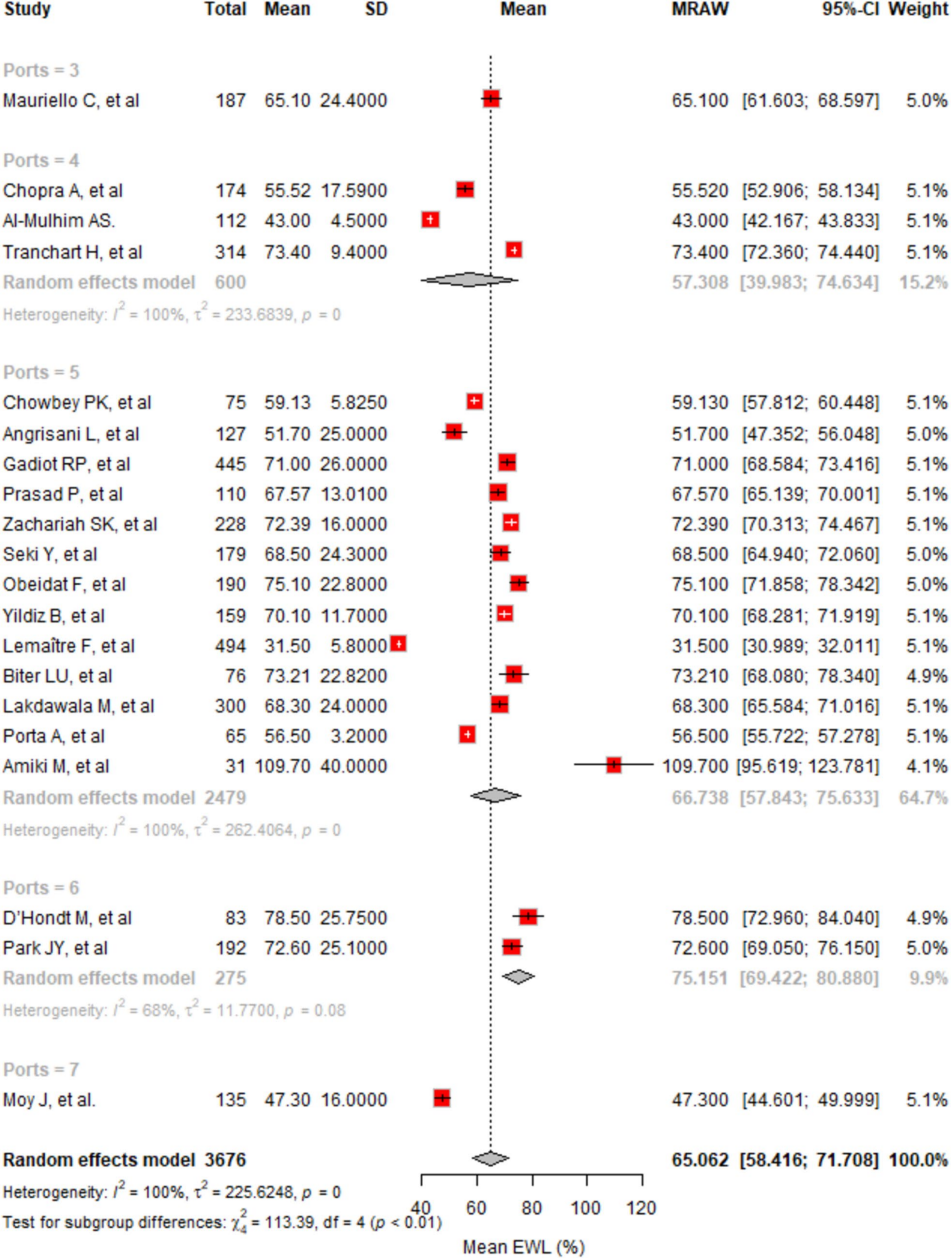
The forest plot illustrates the mean EWL% at 12 months across different studies, segmented by the number of ports used

Across 20 studies and 3676 cases, the mean EWL% was 65.06% (95% CI, 58.42–71.71%) with significant heterogeneity (*I*^2^ = 99.8%) (Fig. 5). Subgroup analysis showed EWL% ranged from 47.30% for 7 ports to 75.15% for 6 ports, with 5-port procedures at 66.74% and 4-port procedures at 57.31%. Variation across port numbers was significant (*p* < 0.0001), and Egger’s test indicated significant asymmetry, suggesting potential publication bias (*p* < 0.0001).

#### EWL% at 24 Months

**Fig. 6:**
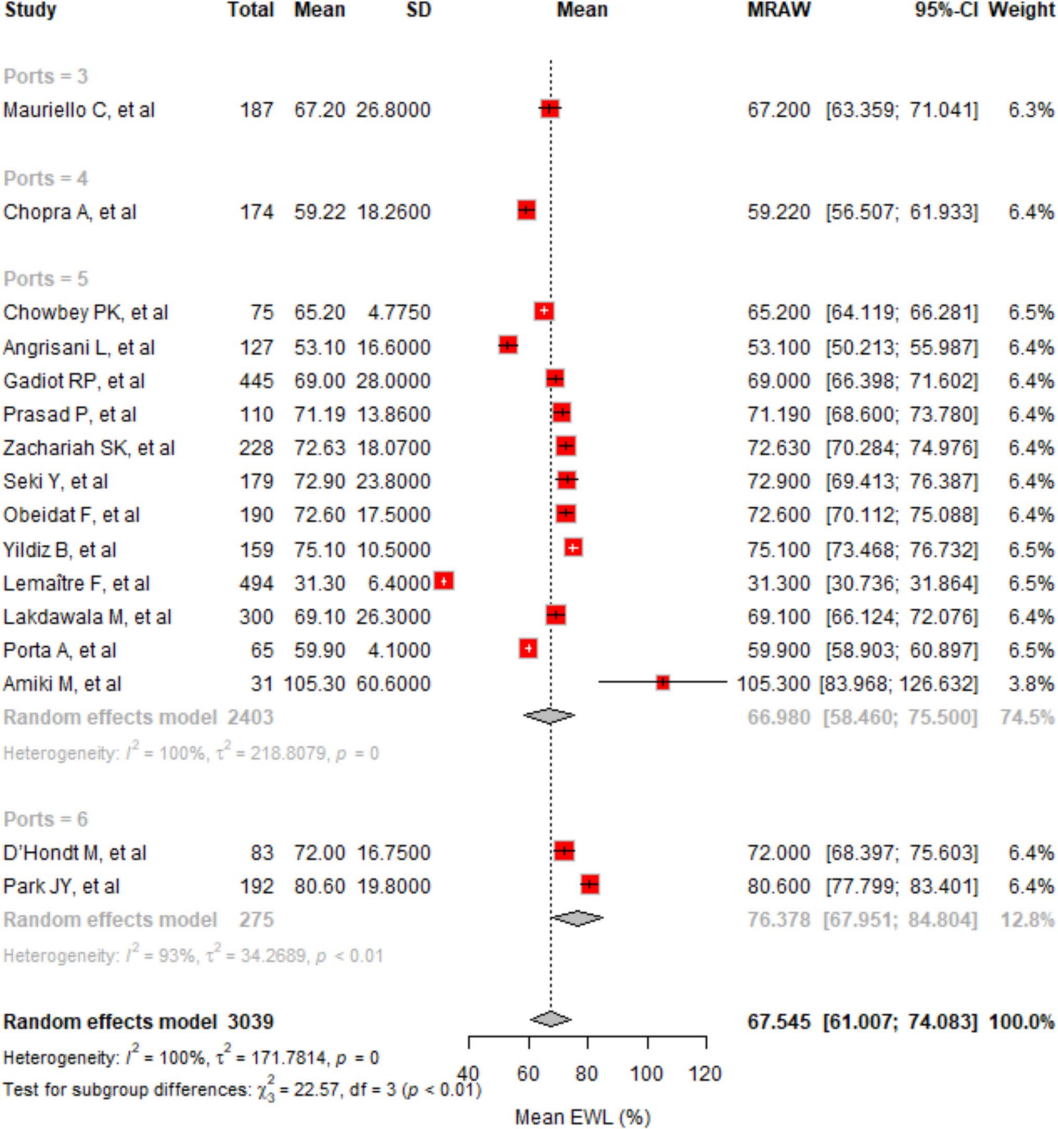
The forest plot illustrates the mean EWL% at 24 months across different studies, categorized by the number of ports used

In 16 studies with 3039 cases, the mean EWL% at 24 months was 67.54% (95% CI, 61.01–74.08%). Heterogeneity was high (*I*^2^ = 99.8%) (Fig. 6). Subgroup EWL% varied, with 76.38% for 6 ports, 66.98% for 5 ports, and 59.22% for 4 ports. Statistical analysis showed significant differences among port numbers (*p* < 0.0001). Egger’s test indicated significant publication bias (*p* < 0.0001).

#### Meta-regression Analysis of EWL% to Different Variables

This meta-regression analysis explores the impact of manipulation angles and port numbers on EWL% at 6, 12, and 24 months after LSG (Table 5). The analyses used mixed-effects models to account for residual heterogeneity and examined the interactions between manipulation angles and the number of ports.

**Table 5:** Meta-regression results for overall operative time to different variables. Azimuth angle 1 = right-sided azimuth angle; azimuth angle 2 = left-sided azimuth angle

Variable	Estimate	SE	*z*-value	*p*-value	95% CI (lower–upper)	Interpretation
BMI	3.78	8.10	0.47	0.64	(− 12.09, 19.65)	Changes in BMI do not significantly affect operative time
Ports	43.58	74.78	0.58	0.56	(− 102.99, 190.14)	Port numbers do not significantly impact operative time
Manipulation angle	− 0.75	1.25	− 0.60	0.55	(− 3.19, 1.70)	Manipulation angles do not significantly impact operative time
Azimuth angle 1	− 0.01	2.85	− 0.00	0.99	(− 5.59, 5.58)	Azimuth angle 1 does not significantly impact operative time
Azimuth angle 2	− 3.35	2.64	− 1.27	0.20	(− 8.52, 1.82)	Azimuth angle 2 does not significantly impact operative time

For EWL% at 6 months, the model revealed substantial residual heterogeneity (*τ*^2^ = 65.34, *I*^2^ = 98.89%), with a high level of unexplained variability. The model explained only 9.80% of the heterogeneity. Neither manipulation angles, port numbers, nor their interaction showed statistically significant effects on EWL%, indicating that these factors may not significantly influence weight loss outcomes in the short term (Table 6).

**Table 6:** Meta-regression results for EWL% and different variables. Azimuth angle 1 = right-sided azimuth angle; azimuth angle 2 = left-sided azimuth angle

Time intervals	Variable	Estimate	SE	*z*-value	*p*-value	95% CI (lower–upper)	Interpretation
EWL% at 6 months interval	Manipulation angle	0.24	0.51	0.46	0.65	− 0.78 to 1.25	Not statistically significant
	Azimuth angle 1 (right side)	− 0.49	1.15	− 0.42	0.67	− 2.76 to 1.77	Not statistically significant
	Azimuth angle 2 (left side)	0.74	0.83	0.89	0.37	− 0.88 to 2.37	Not statistically significant
EWL% at 12 months interval	Manipulation angle	− 0.76	0.97	− 0.78	0.43	− 2.67 to 1.13	Not statistically significant
	Azimuth angle 1	− 2.39	1.98	− 1.2	0.23	− 6.28 to 1.5	Not statistically significant
	Azimuth angle 2	− 0.049	1.53	− 0.32	0.75	− 3.4 to 2.5	Not statistically significant
EWL% at 24 months interval	Manipulation angle	1.24	1.28	0.97	0.33	− 1.27 to 3.76	Not statistically significant
	Azimuth angle 1	1.38	2.82	0.49	0.623	− 4.14 to 6.91	Not statistically significant
	Azimuth angle 2	1.81	2.06	0.88	0.38	− 2.22 to 5.85	Not statistically significant

At 12 months, the model indicated extreme residual heterogeneity (*τ*^2^ = 259.33, *I*^2^ = 99.72%), with no variability accounted for by the model (*R*^2^ = 0.00%). Like the 6-month analysis, manipulation angles, port numbers, and their interaction did not significantly affect EWL%, suggesting that these factors do not have a notable impact on weight loss in the medium term (Table 6).

For EWL% at 24 months, residual heterogeneity remained very high (*τ*^2^ = 184.86, *I*^2^ = 99.56%), and the model again accounted for none of the variability (*R*^2^ = 0.00%). The lack of significant effects for manipulation angles, port numbers, and their interaction suggests that these factors do not contribute substantially to long-term EWL% outcomes (Table 6).

Overall, the meta-regression analyses indicate that manipulation angles and port numbers do not significantly influence EWL% at any of the intervals studied. The high levels of residual heterogeneity suggest that other unexamined factors may play a role in the variability of EWL% outcomes after LSG.

### Meta-regression Analysis of Overall Operative Time vs Different Variables

A mixed-effects meta-regression analysis was conducted to examine the relationship between the number of ports used in LSG and operative time, accounting for BMI variations. Results showed substantial residual heterogeneity (tau^2^ = 1449.83, *I*^2^ = 99.93%), but no significant influence of port number or the BMI-port interaction on operative time (Table 5).

Further analysis of manipulation angles also indicated high residual heterogeneity (tau^2^ = 1244.82, *I*^2^ = 99.88%) with no significant associations found. Similar results were observed for azimuth angles: right-sided (tau^2^ = 1356.59, *I*^2^ = 99.91%) and left-sided (tau^2^ = 1325.99, *I*^2^ = 99.90%), both lacking significant relationships with operative time. Overall, interaction terms between manipulation angles and port numbers were not statistically significant, suggesting these factors do not meaningfully impact operative time in LSG, as summarized in Table 5.

These findings imply that other factors beyond BMI, manipulation angles, and port count may influence operative time, underscoring the need for further research to explore additional variables and better understand the determinants of operative time.

### A New Reporting Checklist Based on Evidential Assessment of the Number of Trocars and Positions

Based on these results, we have developed the PSPOSO reporting checklist, aimed at standardizing the documentation of port site placements in literature. This initiative seeks to provide evidence-based insights into how port site configurations correlate with various outcomes, particularly regarding the operational feasibility and associated complications, with consideration of both the surgical team experience and patient factors.

The PSPOSO checklist serves as a framework for evaluating the efficacy of different trocar placements in laparoscopic MBS. It establishes standardized criteria for documenting port placements, emphasizing critical variables such as port positioning, size, patient orientation, and manipulation angles.

For comprehensive study reporting, the PSPOSO checklist can be found in Table 7. The PSPOSO checklist is designed into both a tailored checklist for individual patients, designed to streamline data collection in alignment with the PSPOSO framework, and a comprehensive checklist for research reporting.

**Table 7:** The proposed PSPOSO checklist. (a) Individualized checklist aiming for the collection of each patient’s data. (b) collective PSPOSO checklist for research reporting

(a) Individualized checklist^a^		(b) Collective PSPOSO reporting checklist	
Variables	Value	Variables	Values
Demographic data		Demographic data	
Patient’s name (optional)			
Age		Mean age	
Sex		Male-to-female ratio	
Initial BMI (kg/m^2^)		Mean initial BMI (kg/m^2^)	
Comorbidities and metabolic disorders	- - -	Comorbidities and metabolic disorders - - -	- *N* (%) - *N* (%) - *N* (%)
Other patient factors		Other patient factors	
Body habitus (e.g., central obesity)	- -	Body habitus (e.g., central obesity)	- *N* (%) - *N* (%)
Visceral fat distribution (optional if preoperative CT was performed)		Visceral fat distribution (optional if preoperative CT was performed)	
Surgeon and surgical team factors		Surgeon and surgical team Factors	
Main surgeons’ height (cm)		Mean of main surgeons’ height (cm)	
**Surgical experience** - Years in practice - Volume of MBS cases annually - Qualifications		**Surgical experience** - Mean years in practice - Mean number of MBS cases annually - Qualifications (*N* (%))	
**Assistant team experience** - Number of assisting surgeons - Qualifications		**Assistant team experience** - Mean number of assisting surgeons - Qualifications (*N* (%))	
Type of procedure and patient position		Type of procedure and patient position	
Type of procedure	-	Type of procedure - - -	- *N* (%) - *N* (%) - *N* (%)
Specific techniques added to the procedure (e.g., omentopexy, gastropexy)	- - -	Specific techniques added to the procedure (e.g., omentopexy, gastropexy)	- *N* (%) - *N* (%) - *N* (%)
Position of the patient (open-leg position or straight-leg position)		Position of the patient - Open-leg position - Straight-leg position	- *N* (%) - *N* (%)
Ports placement and the working angles		Ports placement and the working angles	
**Optical port site (Port 1)** - Site - Distance of Port 1 from the xiphisternum in cm - Size of Port 1 in mm - Lateralization distance of Port 1 from the midline in cm	- - **-** **-**	**Optical port site (Port 1)** Site - - - Mean distance of Port 1 from the xiphisternum in cm Mean size of Port 1 in mm Mean lateralization distance of Port 1 from the midline in cm	- *N* (%) - *N* (%) - *N* (%)
**Right working hand (Port 2)** Site Size of Port 2 in mm R azimuth angle ***anatomical key point***^b^ (right azimuth angle between Port 2 and 1 at a specific anatomical key point) R elevation angle ***anatomical key point***^b^ (angle between the instrument and the patient surface of the instrument of the right hand at a specific anatomical key point)	- **-** **-** **-**	**Right working hand (Port 2)** Site - - - Mean size of Port 2 in mm Mean R azimuth angle ***anatomical key point***^b^ (right azimuth angle between Port 2 and 1 at a specific anatomical key point) Mean R elevation angle ***anatomical key point***^b^ (angle between the instrument and the patient surface of the instrument of the right hand at a specific anatomical key point)	- *N* (%) - *N* (%) - *N* (%)
**Left working hand (Port 3)** Site Size of Port 3 in mm L azimuth angle ***anatomical key point***^b^ (right azimuth angle between Port 2 and 1 at a specific anatomical key point) L elevation angle ***anatomical key point***^b^ (angle between the instrument and the patient surface of the instrument of the right hand at a specific anatomical key point)	- **-** **-** **-**	**Left working hand (Port 3)** Site - - - Mean size of Port 3 in mm Mean L azimuth angle ***anatomical key point***^b^ (left azimuth angle between Port 3 and 1 at a specific anatomical key point) Mean L elevation angle two anatomical ***key point***^b^ (angle between the instrument and the patient surface of the instrument of the left at a specific anatomical key point)	- *N* (%) - *N* (%) - *N* (%)
**Additional ports (Ports 4, 5, 6, 7)** Site Size (mm) Role	- - -	**Additional ports (Ports 4, 5, 6, 7)** Site - - - Mean size (mm) Role	- *N* (%) - *N* (%) - *N* (%)
**Distribution of trocars concerning the optical trocar**	- Triangulation - Sectorization	**Distribution of trocars concerning the optical trocar** Triangulation Sectorization	- *N* (%) - *N* (%)
Identification of intraoperative outcomes and complications		Identification of intraoperative outcomes and complications	
Operative time in mins		Mean operative time in mins	
Intraoperative bleeding in ml		Mean intraoperative bleeding in ml	
Presence of intraoperative findings/complications (e.g., hiatal hernia, GIST)		Presence of intraoperative findings/complications (e.g., hiatal hernia, GIST) - - -	- *N* (%) - *N* (%) - *N* (%)
Postoperative outcomes		Postoperative outcomes	
In-hospital stay (in days)		Mean in-hospital stay (in days)	
Port site complications (if available)		Port site complications - - -	- *N* (%) - *N* (%) - *N* (%)
TWL% at 6 months^c^		Mean TWL% at 6 months^c^	
TWL% at 12 months^c^		Mean TWL% at 12 months^c^	

^a^The individualized checklist aims to assist researchers in collecting data from each patient individually intraoperatively before incorporating it into the PSPOSO checklist

^b^The working angles are calculated based on segmental distribution during the surgical maneuver at key anatomical points, such as the pylorus, incisura, and hiatus. Numerous points can be assessed using various manipulation angles, including azimuth and elevation angles. Adding multiple working angles should add the suffix of the anatomical key point in italics and bold emphasis, e.g., R azimuth angle ***hiatus***, L manipulation angle ***pylorus***

^c^We opted for total weight loss percentage (TWL%) instead of excess weight loss percentage (EWL%) in alignment with the standardized terminology recommended by the IFSO position statement [51]. This choice is intended to support more comprehensive evaluations of port location efficiency and ergonomic standards in relation to clinical outcomes

## Discussion

### The Importance of a Structured Checklist in Surgical Literature

Numerous studies have explored the impact of port placement on surgical outcomes; however, there is a notable scarcity of structured reporting systems addressing these parameters. For example, Shah et al. in their study, “Laparoscopic Sleeve Gastrectomy Smart Trocar Site Modification in Patients with Extreme Obesity” [5], suggested modifications to trocar site placement aimed at enhancing surgical access and maneuverability for patients with extreme obesity (BMI > 60 kg/m^2^). Nevertheless, their report lacked critical details such as the precise positioning of trocar sites, ergonomic considerations, operating theater setup (including distinctions between straight-leg and open-leg positioning), OT table height, monitor positioning, and assessments of operative and suturing times. Moreover, they did not recommend subsequent studies to validate the new technique. These modifications were largely adapted based on clinical experience rather than a systematic, evidence-based methodology. This diminishes the article’s persuasive power for readers seeking a thorough understanding of the new trocar site strategy.

Our analysis of 7173 cases reveals significant variability in port site positioning, sizes, angles of manipulation, and patient positioning documented in the literature. This variation suggests that current practices are predominantly guided by surgeon preference, lacking an evidence-based framework for enhancing or evaluating surgical ergonomics. This underscores the need for future studies to establish standardized, evidence-derived parameters for port placement.

### The Working Angles

Manasnayakorn et al. [47] examined the use of animal models to assess task efficiency and performance quality concerning manipulation angles in laparoscopic surgery. The findings indicated that the optimal manipulation angle fell within the range of 45° to 60°, with correct port placement being crucial to achieve this angle. It was observed that a manipulation angle of 90° resulted in the highest muscle workload on specific muscle groups. Additionally, manipulation angles exceeding 75° were associated with increased difficulty and diminished performance. In addition, achieving equal azimuth angles may be difficult in many practical situations, as unequal azimuth angles are discouraged due to their negative impact on task execution [1].

There exists a direct correlation between the manipulation and the elevation angles. With a manipulation angle of 60°, the optimal elevation angle for achieving the shortest execution time and optimal quality performance is also 60° [48]. Wide manipulation angles require wide elevation angles for optimal performance and task efficiency. When a 30° manipulation angle is determined by the patient’s anatomy or build, the corresponding elevation angle should also be 30° to minimize execution time. For the best ergonomic layout in laparoscopic surgery, a manipulation angle ranging from 45° to 75° with equal azimuth angles is recommended [49].

However, our data contradicted these findings in patients with morbid obesity, showing a mean manipulation angle of 83.05 ± 25.89° and azimuth angles between key ports of 45.32° ± 14.57° for R azimuth and 44.39° ± 18.57° for L azimuth allowed adequate manipulation in MBS. This discrepancy is due to the surgical access in patients with obesity being challenged by the body habitus. However, this discrepancy also highlights the underemphasized value of the working angles, with low reporting rates of these angles at different body habitus and the absence of calculating the elevation angles.

These angles are, however, variable at different points of any MBS, which yields to the difficulty of the reporting system.

### Patient Positioning and Triangulation

Data on patient positioning during LSG showed a predominant use of open-leg positioning (77.2%), which may correlate with better ergonomics and enhanced surgical view. The literature has no current evidence as to whether the straight-leg position can enhance the surgical procedure in MBS, and there are no current comparative studies available.

However, previous studies have assessed both positions in laparoscopic cholecystectomies [50] and showed no significant difference in the angles or in the operative time within an acceptable range between the open-leg and the straight-leg positions. These findings highlight no significant difference with the current data.

### The Number and Position of Ports

The era of conversion from open to laparoscopic MBS procedures watched revolutionary patient outcomes [51, 52]. These outcomes have brought in more patients seeking MBS aiming to optimize the control of adiposity-based chronic diseases. However, with the advancement of the surgical learning curve and the advancement of surgical techniques and technology, more patients were brought in to achieve a healthy lifestyle. This has led surgeons to consider cosmetic approaches to improve a patient’s self-image.

Efforts for establishing a feasible, ergonomic, and yet cosmetic approach for MBS have been taken and discussed in various studies in the literature. With the initial 7-port technique being used in older studies [46] to more innovative and cosmetic techniques such as trans-umbilical single-port SG [7], bikini-line SG [8], and three-port SG [9]. However, the cost of improving cosmesis on the patient’s outcomes between different techniques is questionable.

Jiang et al. [6] conducted a recent meta-analysis to compare the outcomes of single-port SG with conventional LSG, where the conventional group had shown significantly shorter operative time and lower postoperative pain scores. However, EWL%, postoperative development of incisional hernias, and intraoperative bleeding had shown no significant difference between both groups.

In 2017, Abdelbaki et al. introduced an innovative method with 4 ports to undergo LSG [8]. This technique has better cosmetic outcomes, with no evidential comparisons between it and the conventional 5- or 4-port SG. However, the results of the study showed reasonable outcomes in comparison to evidential datasets.

Moreover, more studies have risen to test the safety of lowering the number of trocars from the conventional 5 and 6 trocars to 4 and 3 ports. These claims have driven us to analyze the efficiency of the port numbers to surgical outcomes.

Our analysis revealed that the 4-port method had the longest mean operative time at 90.77 min, while the 3-port method had the shortest at 65.24 min. This difference may be attributed to varying surgical expertise. Despite these discrepancies, the differences in mean operative times across different port configurations were not statistically significant, and we observed high heterogeneity within each subgroup.

However, regarding postoperative EWL%, our data exhibited considerable variability based on the number of ports. At 6 months post-surgery, the EWL% varied significantly, with the 4-port method showing the highest EWL% at 60.86% and the 7-port method showing the lowest at 39.90%. At 12 months, the EWL% was highest for the 6-port subgroup at 75.15% and lowest for the 7-port subgroup at 47.30%. At 24 months, the EWL% was highest for the 6-port method at 76.38% and lowest for the 4-port method at 59.22%. These significant differences between subgroups indicate that the number of ports has a substantial impact on EWL% outcomes. Although the 3-port and 4-port techniques may offer better cosmetic outcomes, they may not offer better surgical outcomes.

### Recent Evidence Raises the Importance of Implicating New Methodological Assessment Models

Recent systematic reviews have evaluated clinical outcomes associated with port site placement in laparoscopic procedures. For instance, Jiang et al. conducted a comparative analysis between single-port laparoscopic sleeve gastrectomy (SLSG) and conventional laparoscopic sleeve gastrectomy (CLSG) [6]. Their study revealed no significant differences in immediate postoperative complications, postoperative weight loss, or late complications; however, SLSG demonstrated a considerably longer operative time. A limitation noted in this review was the heterogeneous approach within the CLSG cohort, characterized by varying port configurations (ranging from 3 to 7 ports) without adequately addressing the implications of port site placement angles on ergonomic factors that could influence patient outcomes.

In a more recent review, Gutiérrez-Ramírez et al. [53] classified laparoscopic sleeve gastrectomy into reduced-port laparoscopic sleeve gastrectomy (RPLSG) utilizing 1–3 ports and conventional port laparoscopic sleeve gastrectomy (CPLSG) employing 4–5 ports. Their findings indicated no statistically significant differences between RPLSG and CPLSG regarding postoperative complications, Clavien-Dindo Classification, and percentage of EWL% at 6, 12, and 24 months, nor in intraoperative duration. Notably, RPLSG was associated with reduced hospital stays. They concluded that while RPLSG is not inferior to CPLSG concerning safety and feasibility for selected patients, the absence of clinically meaningful benefits complicates broader adoption, especially given the ergonomic challenges and the necessity for specialized instruments inherent in these reduced-port techniques.

Despite these insights into various port configurations, there remains a dearth of detailed analysis concerning the ergonomic factors that impact surgical performance and outcome metrics. In contrast, our checklist seeks to fill this void by creating a comprehensive framework aimed at assessing ergonomic parameters, including manipulation angles and azimuth angles. By quantifying these metrics, we can enhance our understanding of the relationship between port placement and surgical efficiency, as well as its effects on surgeon fatigue and patient outcomes.

While reduced-port techniques, such as single-port and three-port laparoscopic surgery, present potential aesthetic advantages, they may introduce significant ergonomic challenges for the surgical team. Our research underscores the imperative of balancing aesthetic considerations with surgical ergonomics to optimize patient outcomes effectively.

Furthermore, by employing the PSPOSO checklist, we aspire to establish a standardized method for reporting port placement, which could facilitate future research endeavors and improve clinical practices. This initiative will empower surgeons to make evidence-based decisions regarding port placement, ultimately enhancing patient outcomes.

### A Standardized Reporting Checklist for Future Research Perspectives

Considering the disparities in research methodologies and the potential for bias in our findings, it is imperative to develop and disseminate a standardized reporting checklist within the International Federation for the Surgery of Obesity and Metabolic Disorders (IFSO) community. This proposed checklist is intended to facilitate a comprehensive evaluation of the advantages and disadvantages associated with the selection of trocar sites in laparoscopic metabolic and bariatric surgeries.

The anticipated benefits of implementing this checklist include standardizing the terminology used to communicate the positioning of trocar sites, providing precise measurements of the manipulation and azimuth angles of port sites, enabling comparative analysis of outcomes and encountered complications for different port site locations, mitigating bias in the reporting of port site locations, and facilitating evaluation of cosmetic outcomes through future self-esteem analysis.

### Port Site Placement and Outcomes for Surgical Obesity and Metabolic Surgeries (PSPOSO) Checklist

The PSPOSO checklist facilitates more accurate comparisons across studies, allowing for benchmarking of outcomes and aiding in the reduction of the learning curve for new surgeons entering the field of bariatric surgery. Additionally, the PSPOSO checklist can provide fundamental data characteristics for studies assessing the postoperative cosmetic outcomes of trocar sites, which can be evaluated using psychiatric parameters such as the body image scale [54–56].

The PSPOSO checklist, although primarily developed based on the evidence surrounding trocar placement in LSG, applies to a range of metabolic and bariatric surgery (MBS) procedures. This checklist encompasses several domains, such as demographic characteristics of study participants, the positioning and angles of the ports during surgical dissection at the hiatus—specifically measuring azimuth, manipulation, and elevation angles—the spatial distribution of trocars relative to the optical trocar, and the patient’s positioning.

### Integrating Ergonomic Variability to Ascertain Clinical Outcomes in the PSPOSO Checklist

Ergonomic variability during surgical procedures is influenced by factors such as the surgeon’s height, instrument strain, level of experience, patient body habitus, and the working angles required at different stages of the operation. In our study, we determined the working angles using the hiatus as a fixed reference point, as measuring these angles at multiple stages of the sleeve gastrectomy was not feasible as the variability in the sizes and shapes of the stomach across patients was not mentioned in any of the studies extracted. This limitation, outlined in our methodology, underscores the challenges of assessing segmental angle measurements in existing studies where stomach anatomy was not consistently documented.

Recognizing the significant impact of the stomach’s non-linear shape on ergonomics—particularly during critical phases like the initial stapling—we acknowledge the need for more granular analysis of working angles at key anatomical landmarks such as the hiatus, the angular incisura, and the pyloric sphincter. To address this gap, our checklist incorporates provisions for segmental angle measurements at these critical points. By enabling future studies to assess the ergonomic implications of the initial stapling angle and other procedure-specific factors, we aim to foster a deeper understanding of how anatomical variability influences clinical outcomes in laparoscopic sleeve gastrectomy.

Additionally, the PSPOSO checklist integrates the experience levels of the surgical team and other patient factors to mitigate confounding variables that may adversely affect surgical outcomes. In addition to the patient’s BMI and demographic profile, the checklist encompasses a range of pertinent patient variables such as metabolic conditions (including thyroid disorders, immunological diseases, and diabetes mellitus), body habitus (e.g., central obesity), and characteristics indicative of a complex abdomen, such as the presence of abdominal wall hernias or previous major open abdominal surgery scars [57–59].

The surgical team’s experience is assessed through the number of assisting personnel and their surgical qualifications [60], alongside the lead surgeon’s expertise, evaluated in terms of years of experience in MBS and the volume of MBS cases conducted annually. Identifying body and metabolic characteristics and surgical team experience can highlight various heterogeneous data (as presented in our study) and show less bias in defining feasibility and surgical outcomes.

Instrumental strain during laparoscopic procedures is influenced by the surgeon’s height, as it affects the alignment between the surgeon’s reach and the operative field [61]. Variations in height can lead to increased muscle strain, particularly in the arms and lower back, during prolonged surgeries [61]. Studies highlight that inadequate ergonomic adjustments for height exacerbate fatigue, impacting both efficiency and physical well-being.

The PSPOSO checklist addresses this by emphasizing the documentation of surgeon height in relation to working angles, aiming to better understand its impact on outcomes and develop strategies to optimize ergonomic practices.

Outcomes derived from the checklist include an assessment of the technique’s feasibility, quantified through operative time, blood loss, and late postoperative results, such as length of hospital stay, TWL%, and port site complications. Additionally, factors that could prolong the operative time and affect the surgical outcomes, such as the utilization of specific surgical techniques—omental reduction [62], gastropexy, or omentopexy [63]—incidental findings [64, 65], and intraoperative complications [66], should be included in the checklist.

We opted for TWL% instead of excess weight loss percentage (EWL%) in alignment with the standardized terminology recommended by the IFSO position statement [51]. This choice is intended to support more comprehensive evaluations of port location efficiency and ergonomic standards in relation to clinical outcomes.

### Weighting Ergonomic Difficulties in the Utilization of Robotic MBS

The escalating technical demands for robotic surgeries point to a paradigm shift in surgical practice. Robotic surgery aims to mitigate the ergonomic challenges associated with traditional open and laparoscopic methodologies, albeit introducing new obstacles in the process [67].

A notable ergonomic concern in robotic surgery is the risk of suboptimal postures, particularly with improper usage of armrests, which can lead to discomfort and potential musculoskeletal strain [67]. Moreover, the requirement for extended usage of 3D visualization systems in certain robotic frameworks may exacerbate ergonomic concerns for the surgeon. Although robotic surgery can reduce physical strain compared to conventional laparoscopic techniques, it is imperative to critically assess the specific design features of each robotic platform [67]. The integration of appropriate ergonomic practices is essential to mitigate the risk of musculoskeletal disorders and safeguard the long-term health of surgical teams.

Despite the potential benefits of robotic surgeries in MBS, including efficiency, safety, and enhanced cosmetic results, current evidence suggests that robotic MBS yields outcomes comparable to traditional laparoscopic MBS [68]. Notably, robotic MBS demonstrates a distinct advantage in achieving superior cosmetic outcomes, particularly with single-port techniques when compared to multiport robotic systems [69]. This differentiation could incentivize surgeons to adopt robotic methodologies to both replicate traditional outcomes and enhance patient satisfaction through improved aesthetic results.

However, it is crucial to consider the significant cost implications associated with robotic surgical systems compared to laparoscopic alternatives, which raises pertinent questions regarding their feasibility for routine application in MBS. Consequently, while robotic surgery signifies a noteworthy progression in surgical innovation and cosmetic appeal, a rigorous examination of its ergonomic challenges and cost-effectiveness is paramount for its sustainable incorporation into MBS clinical practice.

Thus, it is paramount to still consider laparoscopic approaches and improve patient outcomes by enhancing their ergonomic feasibility.

### Strengths and Limitations

This systematic review and meta-analysis possess several notable strengths, notably the identification of 7022 pertinent publications on laparoscopic sleeve gastrectomy (LSG) across four primary databases, culminating in the selection of 34 relevant studies that encompass a total of 7173 cases for analysis, all conducted in adherence to PRISMA guidelines.

We introduce a novel framework, PSPOSO (Port Site Placement and Outcomes for Surgical Obesity and Metabolic Surgeries), that is underpinned by evidence-based principles regarding port placement, manipulation angles, and patient positioning. This framework serves as a straightforward and rapid assessment checklist, designed to standardize port placement practices across diverse clinical settings. The aim is to mitigate variability in minimally invasive bariatric surgery (MBS), enhance clarity in comparative outcome analyses, and ensure consistent reporting of critical surgical parameters. Additionally, we utilized ImageJ to extract angles pertinent to the surgical procedure, enabling us to estimate manipulation angles effectively. The review also provides an in-depth analysis of how port placement influences outcomes in LSG.

Despite these strengths, the study acknowledges several limitations. Primarily, the data is sourced from previously conducted studies that employed heterogeneous methodologies and objectives. Furthermore, while our sample size is substantial, it may not comprehensively reflect global surgical practices, given the variations in techniques and patient demographics encountered internationally.

## Conclusion

This study highlights the variability in port site placement, sizes, and manipulation angles during LSG and their potential impact on patient outcomes. Overall, the meta-regression analyses indicate that manipulation angles and port numbers do not significantly influence EWL% at any of the intervals studied. The high levels of residual heterogeneity suggest that other unexamined factors may play a role in the variability of EWL% outcomes after LSG. Moreover, it implies that other factors beyond BMI, manipulation angles, and port count may influence operative time, underscoring the need for further research to explore additional variables and better understand the determinants of operative time.

The Port Site Placement and Outcomes for surgical obesity and Metabolic Surgeries (PSPOSO) checklist (designed into both a tailored checklist for individual patients and a comprehensive checklist for research reporting) is a structured framework to standardize these variables, laying the groundwork for enhanced reproducibility and outcome comparisons.

### Recommendation and Further Research

This study highlights the urgent need for standardized, evidence-based criteria to enhance comparability and reproducibility in minimally invasive surgery (MIS). The absence of a universally accepted language regarding trocar site placement has led to significant variability across literature, particularly in ergonomic considerations and key outcomes such as operative time and complication rates.

We advocate for the implementation of the PSPOSO checklist across all bariatric procedures to foster improved patient safety and surgical practices. The adoption of this framework will help establish consistent, high-quality care and standardized reporting for metabolic surgeries. To validate its effectiveness, it is essential to apply the checklist in diverse global settings, encompassing a wide range of surgical techniques and patient demographics.

Future research should focus on prospective studies that implement and assess the PSPOSO checklist in real time, aiming to standardize techniques and enhance outcomes across varied patient populations. Additionally, further investigation into specific angles and port placements is necessary to understand their influence on postoperative complications, thus optimizing MIS practices.

## Supplementary Information

Below is the link to the electronic supplementary material.

Supplementary file1 (DOCX 183 KB)

## Data Availability

No datasets were generated or analysed during the current study.

## Abbreviations

*BMI*: Body mass index
*CLSG*: Conventional laparoscopic sleeve gastrectomy
*CPLSG*: Conventional port laparoscopic sleeve gastrectomy
*EWL%*: Excess weight loss percentage
*IFSO*: International Federation for the Surgery of Obesity and Metabolic Disorders
*LSG*: Laparoscopic sleeve gastrectomy
*MBS*: Metabolic and bariatric surgeries
*MIS*: Minimally invasive surgery
*NOS*: Newcastle-Ottawa Scale
*OT*: Operating table
*PSPOSO*: Port Site Placement and Outcomes for Surgical Obesity and Metabolic Surgeries
*RPLSG*: Reduced-port laparoscopic sleeve gastrectomy
*SG*: Sleeve gastrectomy
*SLSG*: Single-port laparoscopic sleeve gastrectomy
*TWL%*: Total weight loss percentage
