# Supplementary material for: Port Site Placement and Outcomes for Surgical Obesity and Metabolic Surgeries (PSPOSO) Checklist: A New Reporting Checklist Based on Evidential Assessment of the Number of Trocars and Positions
Source: Obes Surg. 2025 Feb 4;35(3):1086–108. doi: 10.1007/s11695-025-07694-y (PMC11906533; doi:10.1007/s11695-025-07694-y)
Supplement: Supplementary file 1 — Supplementary file1 (DOCX 183 KB) [file 11695_2025_7694_MOESM1_ESM.docx]

# Supplementary File 1

# Methodological estimation of the angles


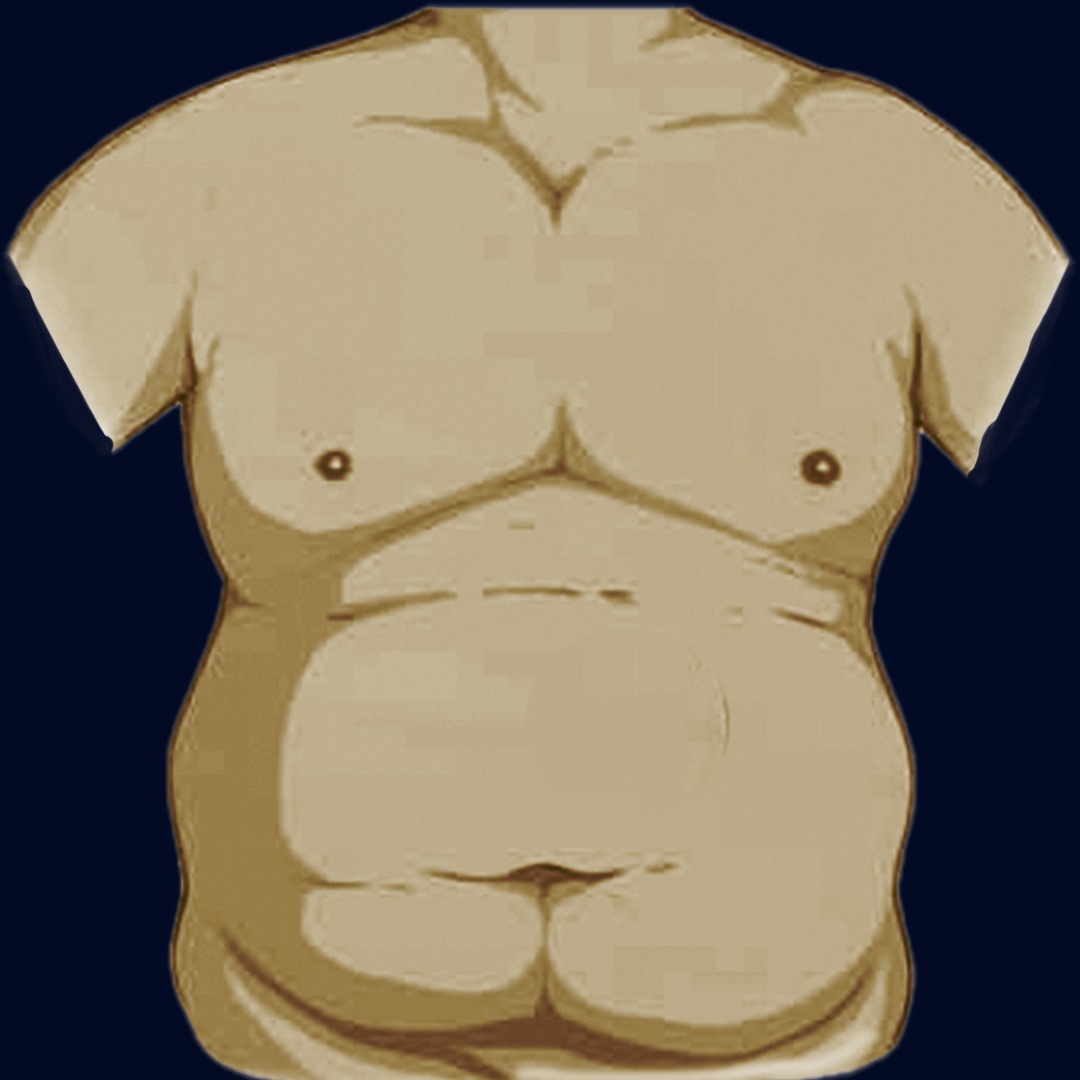


Figure S1: Template used for identifying the positions of the trocars according to each study


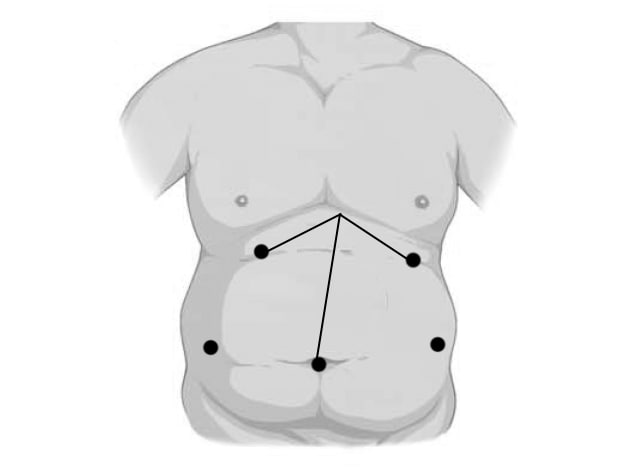


Figure S2: Example 1 for setting the trocars (points) and the working angles meeting at the surface anatomy of the hiatus


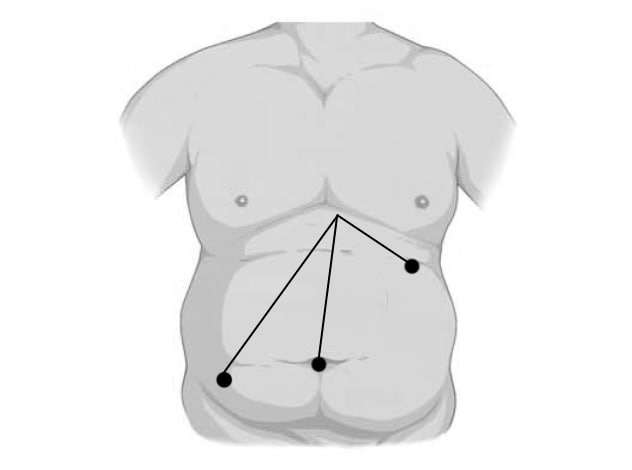


Figure S3: another example of the hiatus being set for the meeting point in another study

# Results Supplementary Figures and Tables

**Table S1: mean EWL% at 6, 12, and 24 months of the studies included in the Systematic Review**

| **Post-operative data** | **Mean ± SD** |
| --- | --- |
| **EWL % at 6 months**  Min - Max  Mean ± SD | 25.40 - 100.0  52.87 ± 8.46 |
| **EWL % at 12 months**  Min - Max  Mean ± SD | 22.50 - 160.0  64.53 ± 15.84 |
| **EWL % at 24 months**  Min - Max  Mean ± SD | 23.0 - 173.0  68.12 ± 13.76 |


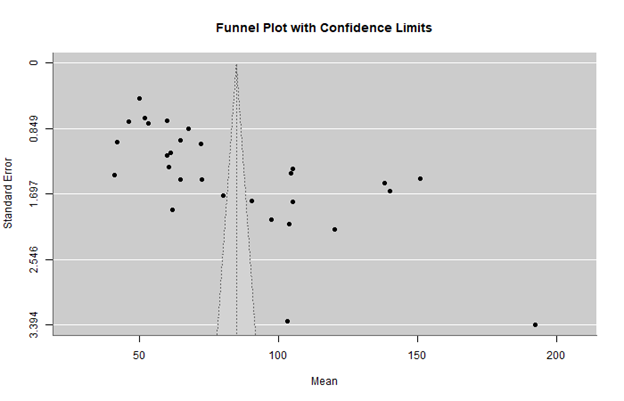


Figure S4: Operative time Funnel plot the regression test for funnel plot asymmetry indicates significant asymmetry (t = 5.23, df = 27, p < .0001), suggesting potential publication bias in the meta-analysis.


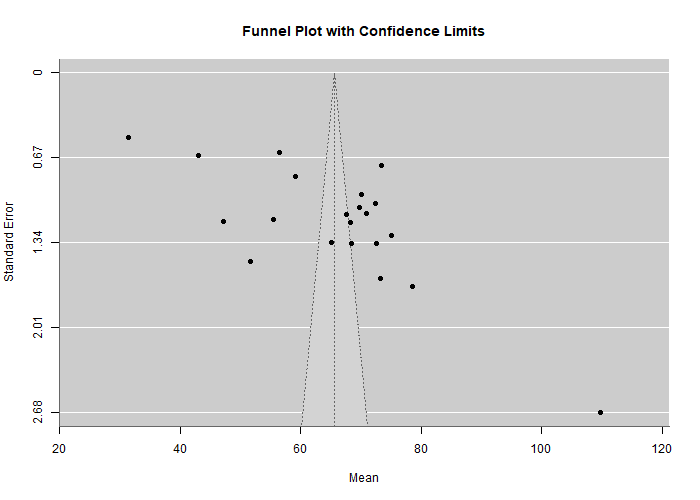


Figure S5: The regression test for funnel plot asymmetry for EWL% at 12 months reveals significant asymmetry (t = 4.2311, df = 19, p = 0.0005), indicating the presence of potential publication bias


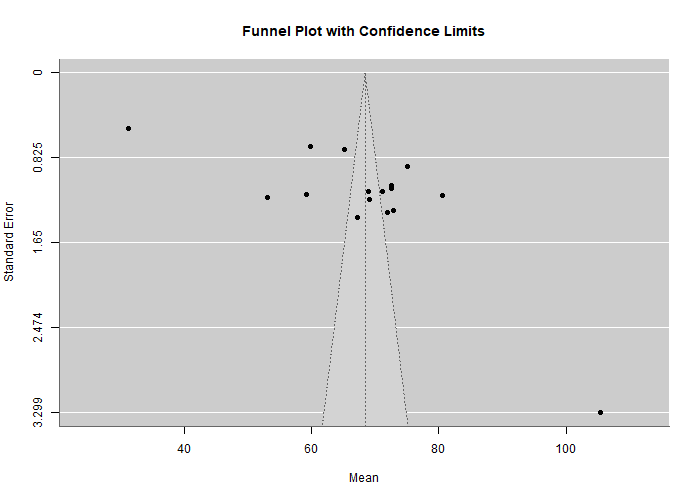


Figure S6: Funnel plot for EWL% at 24 months: The test results indicate significant asymmetry (t = 4.2311, df = 19, p = 0.0005), suggesting potential publication bias in the meta-analysis. The limit estimate for the effect size as the standard error approaches zero is b = 28.8481, with a 95% confidence interval of (13.6758, 44.0204). This implies that the true effect size may be meaningful, highlighting the need to interpret the findings considering potential bias carefully.
